# MicroRNA-181a as novel liquid biopsy marker of central nervous system involvement in pediatric acute lymphoblastic leukemia

**DOI:** 10.1186/s12967-020-02415-8

**Published:** 2020-06-22

**Authors:** Bálint Egyed, Nóra Kutszegi, Judit C. Sági, András Gézsi, Andrea Rzepiel, Tamás Visnovitz, Péter Lőrincz, Judit Müller, Marianna Zombori, Csaba Szalai, Dániel J. Erdélyi, Gábor T. Kovács, Ágnes F. Semsei

**Affiliations:** 1 2nd Department of Pediatrics, Semmelweis University, 7-9 Tűzoltó Str, Budapest, 1094 Hungary; 2 Department of Genetics, Cell- and Immunobiology, Semmelweis University, 4 Nagyvárad Sqr, Budapest, 1089 Hungary; 3 Department of Anatomy, Cell and Developmental Biology, Eötvös Loránd University, 1/c Pázmány Promenade, Budapest, 1117 Hungary; 4 Heim Pal National Pediatric Institute, 86 Üllői Str, Budapest, 1089 Hungary; 5 MTA-SE Immune-Proteogenomics Extracellular Vesicle Research Group, Semmelweis University, 4 Nagyvárad Sqr, Budapest, 1089 Hungary; 6 Department of Measurements and Information Systems, Budapest University of Technology and Economics, 2 Magyar tudosok korutja, Budapest, 1117 Hungary

**Keywords:** Central nervous system involvement, Pediatric leukemia, Liquid biopsy, microRNA-based biomarker, Small extracellular vesicles

## Abstract

**Background:**

Refractory central nervous system (CNS) involvement is among the major causes of therapy failure in childhood acute leukemia. Applying contemporary diagnostic methods, CNS disease is often underdiagnosed. To explore more sensitive and less invasive CNS status indicators, we examined microRNA (miR) expressions and extracellular vesicle (EV) characteristics.

**Methods:**

In an acute lymphoblastic leukemia (ALL) discovery cohort, 47 miRs were screened using Custom TaqMan Advanced Low-Density Array gene expression cards. As a validation step, a candidate miR family was further scrutinized with TaqMan Advanced miRNA Assays on serial cerebrospinal fluid (CSF), bone marrow (BM) and peripheral blood samples with different acute leukemia subtypes. Furthermore, small EV-rich fractions were isolated from CSF and the samples were processed for immunoelectron microscopy with anti-CD63 and anti-CD81 antibodies, simultaneously.

**Results:**

Regarding the discovery study, principal component analysis identified the role of miR-181-family (miR-181a-5p, miR-181b-5p, miR-181c-5p) in clustering CNS-positive (CNS^+^) and CNS-negative (CNS^‒^) CSF samples. We were able to validate miR-181a expression differences: it was about 52 times higher in CSF samples of CNS^+^ ALL patients compared to CNS^‒^ cases (n = 8 vs. n = 10, ΔFC = 52.30, p = 1.5E−4), and CNS^+^ precursor B cell subgroup also had ninefold higher miR-181a levels in their BM (p = 0.04). The sensitivity of CSF miR-181a measurement in ALL highly exceeded those of conventional cytospin in the initial diagnosis of CNS leukemia (90% vs. 54.5%). Pellet resulting from ultracentrifugation of CNS^+^ CSF samples of ALL patients showed atypical CD63^−^/CD81^−^ small EVs in high density by immunoelectron microscopy.

**Conclusions:**

After validating in extensive cohorts, quantification of miR-181a or a specific EV subtype might provide novel tools to monitor CNS disease course and further adjust CNS-directed therapy in pediatric ALL.

## Background

Despite extensive endeavors towards risk-directed treatment, acute leukemia remain the major cause of death and is responsible for the highest number of disability-adjusted life-years in children and adolescents with cancer [1]. Leukemic involvement of the central nervous system (CNS) represents an outstanding therapeutic problem [2]. Prophylactic regimens (intrathecal and high-dose systemic chemotherapy, cranial irradiation) against CNS leukemia has become a prerequisite of successful treatment: prior to the introduction of CNS-targeted therapy in the 1960s, symptomatic meningeal leukemia developed in more than 50% of cases and CNS relapse rate was over 65% in childhood acute lymphoblastic leukemia (ALL) [3, 4]. Nevertheless, patients with ALL who have a history of overt CNS involvement at the initial diagnosis are still susceptible to relapse with only 15–20% 5-year survival rates [5]. CNS disease in acute myeloid leukemia (AML) entails poor outcomes, and optimal treatment has not been established, mainly due to the rarity of this condition [6].

According to the modern approach, the CNS invasion can be the ultimate step of the tumor cascade [7], and vice versa, CNS relapse of acute leukemia constitutes an early manifestation of systemic relapse and subclones freely traffic between leukemic niches of different organs [8]. Thus, neural and bone marrow (BM) compartments also need to be monitored to reach proper disease control. Very sensitive laboratory methods are available to monitor minimal residual disease in the BM. Currently used tests can quantify the leukemic cells after a fall by 4 to 6 logs [9]. We lack tools with similar sensitivity regarding the CNS compartment. The most widespread contemporary methods for the assessment of cerebrospinal fluid (CSF) are conventional cytologic examination of a cytocentrifuge smear and flow cytometry [10]. Especially due to the paucity of cells in the CSF, both methods yield a high proportion (more than 40%) of false-negative reports among patients with proven CNS infiltration by neuroimaging or autopsy [11]. Consequently, a gold standard method for staging the CNS involvement in acute leukemia has not been established yet.

We hypothesize that some non-cellular biomarkers could identify undiagnosed CNS leukemia cases and unravel treatment response follow-up in this important compartment. MicroRNAs (miRs) belong to the class of highly conserved small non-coding RNAs that play key regulatory role in a wide range of biological processes such as proliferation, differentiation and survival [12]. Altered expression patterns of miRs have been increasingly recognized associating with progression in various types of tumors including acute leukemia [13]. The deregulation of miRs in disease conditions can be harnessed as potential new-generation therapeutics.

Cells in the human body continuously discharge large amounts of vesicles 30–1000 nm in diameter into the extracellular space. Extracellular vesicles (EVs) are important factors of intercellular communication: they transfer DNA, coding and regulatory RNAs, lipids and proteins in the microenvironment as well as among very distant tissues [14]. It is widely accepted that EV production increases in the tumor microenvironment, hence, these particles may facilitate proper “cancer cell biopsy”. In studies performed on different leukemia patients, presence of EVs originating from leukemic cells was shown and certain alterations in the EVs correlated with the hijacking of disease niches and treatment efficacies [15].

In this study, we provide an analysis of miRs as putative biomarkers of pediatric acute leukemia stage and dynamics in the CNS compartment and a novel proposal of examining small EVs in the CSF of CNS leukemia patients. We based our miR investigation not only on CSF but also on bone marrow (BM) and peripheral blood (PB) samples, bearing in mind the aim of developing less invasive diagnostics.

## Materials and methods

### Patient cohorts and samples

Between October 2015 and August 2019, we collected peripheral blood, bone marrow (PB and BM, respectively; in tubes with 3.2% buffered sodium citrate solution) and CSF (native) from 186 consecutive, unselected patients (aged ≤ 18 years) diagnosed with acute leukemia or concomitant relapse in two Hungarian pediatric hematology centers (Semmelweis University 2nd Department of Pediatrics and Heim Pal National Pediatric Institute). All patients were enrolled into a Berlin-Frankfurt-Münster (BFM) trial, and samples were obtained at different timepoints in the 1st month of chemotherapy. Patients underwent diagnostic and follow-up lumbar puncture (LP). CSF was examined in the participating hospital according to BFM protocols with the use of a cell counting chamber and conventional cytology after cytocentrifugation. For the present study, CNS status was defined as follows, with respect to subsequent rate of relapses [16]: CNS-involved (CNS^+^; nontraumatic LP with unambiguously identified blasts on cytospin preparation of CSF) and CNS-negative (CNS^−^; blast-free CSF). CNS^−^ cases were selected based on conventional cytologic preparations double-checked by expert pediatric hematologists (comments in Table 1) and the absence of meningeal infiltration signs by medical imaging and/or clinical neurologic examination. Patients with traumatic LP were excluded. Preparation of platelet-free plasma (PFP) from PB and BM samples was carried out within 2 h of sampling by centrifugation at 2500*g* for 15 min two times [17].

**Table 1:** Clinical characteristics of childhood acute leukaemia patients involved in discovery and validation cohorts

Total number of examined samples			Samples			
			Discovery cohort	Validation cohort		
			20	138		
Control group (sample type, number of individuals)			PB, n = 10	CSF, n = 6		
ID	Diagnosis, treatment	CSF cytology (CNS status according to protocol)	CSF	CSF	BM	PB
CNS+ patient group						
P1	ALL, late-onset Rel^b^	lymphoid cells with unambiguous blasts (CNS-2)	d0	d0, d15	d0, d15, d30	d0, d15, d30
P2	ALL, de novo^c^	86 WBCs; high amount of blasts (CNS-3)	d0, d15	d0, d15, d32, d45	d37	d0, d37
P3	ALL, de novo^a^	1 unambiguous blast, 2 suspicious cells (CNS-2)	d0	d0, d15, d33	d0, d15, d33	d0, d15, d33
P4	ALL, de novo^a^	4 WBCs/μl with blasts (CNS-2)	d0, d15	‒	‒	‒
P5	ALL, de novo^a^	18 lymphoid cells (mainly blasts), 20 Gumprecht shadows (CNS-2)	‒	d0, d15	d0, d15	d0, d15
P6	ALL, de novo^a^	1 unambiguous blast (CNS-2)	‒	d0, d15, d33	d0, d15, d33	d0, d15, d33
P7	MPAL, de novo^a^	some cells, 70% blast (CNS-2)	‒	d15, d33	d0, d15, d33	d0, d15, d33
P8	AML, de novo^d^	53 cells; 8% blast (CNS-2)	‒	d0, d15	d0	d0, d15
P9	AML, de novo^d^	7 WBCs/μl; numerous blasts (CNS-3)	‒	d0, d28	d0, d28	d0, d28
P10	ALL, early-onset Rel^b^	43 WBCs, mostly blasts (CNS-3)	‒	d0	d0	‒
P11	ALL, de novo^a^	nucleated cells, 70% blast (CNS-2)	‒	d0, d15	‒	‒
P12	ALL, de novo^a^	35 lymphoid cells with 10% blast (CNS-2)	‒	d0, d15, d26, d40	‒	‒
CNS^−^ patient group						
P13	ALL, de novo^a^	1 cell/μl without blasts (CNS-1)	d0	‒	‒	‒
P14	ALL, de novo^a^	without cells (CNS-1)	d0	‒	‒	‒
P15	ALL, de novo^a^	without cells (CNS-1)	d0	‒	‒	‒
P16	ALL, de novo^a^	1 cell/μl without blasts (CNS-1)	d0	‒	‒	‒
P17	ALL, early-onset Rel^b^	squamous cells, 2 lymphocyte, 1 Gumprecht shadow (CNS-1)	‒	d0, d15	d0, d15, d37	d0, d15, d37
P18	ALL, early-onset Rel^b^	without cells (CNS-1)	‒	d0, d15	d0, d15	d0, d15
P19	ALL, de novo^a^	without cells (CNS-1)	‒	d0, d15, d33	d0, d15, d33	d0, d15, d33
P20	ALL, de novo^a^	only a few RBCs (CNS-1)	‒	d0, d15	d0, d15, d33	d0, d15, d33
P21	ALL, de novo^a^	2 cells without blasts (CNS-1)	‒	d0, d15, d33	d0, d15, d33	d0, d15, d33
P22	MPAL, de novo^a^	a few squamous cells on cytospin (CNS-1)	‒	d15, d38	d0, d15, d38	d0, d15, d38
P23	AML, de novo^d^	some RBCs without blasts (CNS-1)	‒	d0	d0, d23	d0, d15, d23
P24	AML, de novo^d^	1 segmented, 2 band neutrophil granulocytes (CNS-1)	‒	d0, d28	d0, d15, d28	d28
P25	ALL, de novo^a^	1 segment, 8 WBC (CNS-1)	‒	d0	d0	‒
P26	ALL, de novo^a^	without cells (CNS-1)	‒	d0	‒	‒
P27	ALL, de novo^a^	without cells (CNS-1)	‒	d0	‒	‒
P28	ALL, de novo^a^	4 segmented neutrophil cells, 3 lymphocytes, 1 monocyte, 1 Gumprecht shadow, but no blasts (CNS-1)	‒	d0	‒	‒
P29	ALL, de novo^a^	without cells (CNS-1)	‒	d0	‒	‒

Treatment guidelines: ^a^ALL IC BFM 2009, ^b^ALL REZ BFM 2002, ^c^Interfant 2006, ^d^AML BFM 98

*CNS* central nervous system, *ALL* acute lymphoblastic leukaemia, *AML* acute myeloid leukaemia, *MPAL* mixed phenotype acute leukaemia, *Rel* relapse, *CSF* cerebrospinal fluid, *BM* bone marrow, *PB* peripheral blood, *WBC* white blood cell, *RBC* red blood cell, *d* day

To constitute a discovery cohort, we selected CNS^+^ ALL patients (n = 4) and matched CNS^−^ patients at a ratio of 1:1 according to sex, age at diagnosis and immunophenotype (Table 1 and Additional file 1: Table S1). Related CSF and PB samples were analyzed. Reference PB samples were obtained from individuals with non-malignant diseases (n = 10; median age: 4.2; the proportion of males: 0.4; diagnoses: vitamin D deficiency, impetigo, otitis media, latent hypothyroidism, juvenile idiopathic arthritis, iron deficiency anemia, neurofibromatosis type 1, phimosis). Validation cohort consisted of ALL, AML or mixed phenotype acute leukemia (MPAL) patients with CNS^+^ (n = 11) and CNS^‒^ (n = 13) diseases (Table 1 and Additional file 1: Table S1). In this cohort, median follow-up time of patients after involving them into this study was 1.4 years (range 0.1‒3.3). Analysis was performed on CSF, BM and PB samples. Reference CSF samples in the validation study were derived from spinal muscular atrophy (SMA) patients (n = 6, median age: 1.34, proportion of males: 0.8).

### RNA isolation and cDNA amplification

Total RNA was isolated from BM PFP, PB PFP and human native CSF samples using the miRNeasy Serum/Plasma Mini Kit (Qiagen, Hilden, Germany), according to the manufacturer’s instructions with an initial volume of 100 μl. RNA was eluted into 12 μl RNase-free water and 1.2 μl RNase Inhibitor (Thermo Fisher Scientific, Waltham, MA, USA) was added per sample, then stored at − 80 °C. Synthesis of cDNA by universal reverse transcription chemistry after 3′ poly-A tailing and 5′ ligation was performed with a TaqMan Advanced miRNA cDNA Synthesis Kit (Thermo Fisher Scientific, Waltham, MA, USA) following the manufacturer’s guidelines. To improve the detection of low-expressing miR targets, the cDNA was then amplified using universal miR-Amp primers of the latter kit. Nucleic acid concentration was measured by NanoDrop 1000 Spectrophotometer (Thermo Fisher Scientific, Waltham, MA, USA).

### MiR expression measurements in discovery and validation cohorts

A selection of potential miR marker candidates for CNS leukemia was obtained by compiling miRs expressed in (i) BM cells under leukemic conditions, (ii) blast cells with distinct genetic features and (iii) human platelets to rule out sample mistreatment, as previously described [18]. This process resulted in 47 candidate miRs selected for profiling, see Additional file 1: Table S2. The expression of candidates was measured by quantitative polymerase chain reactions (qPCR) performed on Custom TaqMan Advanced Low-Density Array (TLDA) microfluidic cards (Thermo Fisher Scientific, Waltham, MA, USA), according to the manufacturer’s description using QuantStudio 7 Flex Real-Time PCR System (Thermo Fisher Scientific, Waltham, MA, USA). As a second step, identified differentially expressed (CNS^+^ vs. CNS^‒^ groups) miR candidates were assessed in the validation cohort. This qPCR verification was carried out on the 7900HT Fast Real-Time PCR System (Thermo Fisher Scientific, Waltham, MA, USA). The experiments were performed in duplicates using TaqMan Advanced miRNA Assays and TaqMan Fast Advanced Master Mix (Thermo Fisher Scientific, Waltham, MA, USA) following the manufacturer’s instructions.

Cel-miR-39 (synthesized with 5′-phosphates; Assay ID: 000200) was added as a non-human spike-in control to samples during RNA isolation. After the qPCR reactions, all amplification curves were inspected regardless to patients’ CNS status in order to exclude nonspecific artefacts. The expression fold change value was calculated according to the comparative cycle threshold (Ct) algorithm for each sample. In the validation analysis, at each miR under the detection level, missing Ct values were replaced with maximum Ct per assay, except for normalizer miR.

### Determination of CSF EV characteristics

To evaluate small EV density, firstly, CD63^+^ EVs were isolated from CSF samples after centrifuging at 300*g* for 5 min and 2000*g* for 20 min. EVs were bound by adding 10 μl anti-CD63-coated beads (Exosome-Human CD63 Isolation/Detection Reagent, Thermo Fisher Scientific, Waltham, MA, USA) prepared according to the manufacturer’s instructions to 300 μl supernatant. After overnight incubation with mixing at 4 °C, beads were magnetically separated (DynaMag Spin Magnet, Thermo Fisher Scientific, Waltham, MA, USA), washed with PBS and beads were labelled with anti-CD63-PE (SAB4700218, Sigma-Aldrich) for 20 min. Up to 5000 beads were then measured on a FACSCalibur (BD Biosciences, San Jose, CA, USA) instrument. Output was analyzed using FlowJo software (FlowJo LLC, Ashland, OR, USA; version: 10.5.3).

In another experiment, small EV-rich fractions (irrespective of the presence of CD63) were isolated for further analysis according to the directives of the International Society for Extracellular Vesicles [19]. Briefly, CSF was centrifuged in order to remove large cell debris (300*g* for 5 min) and pelleting of large, secreted microvesicles from the cell-free supernatant (12,500*g* for 20 min). A collection of small EVs was performed with two rounds of ultracentrifugation (UC) at 100,000*g* for 70 min at 4 °C, completed with a washing step using PBS between the UCs. Finally, the pellet was resuspended in 60 µl PBS. A 3 µl droplet was dried on a formvar-coated 300 mesh Ni grid (SPI Supplies, USA) for 10 min. The residual liquid was removed and samples were processed for immunoelectron microscopy with anti-CD63 and anti-CD81 antibodies, simultaneously, following previously described steps [20]. Sample examination was carried out using JEOL 1011 transmission electron microscope (Tokyo, Japan).

### Bioinformatic analysis

The R language and environment for statistical computing and graphics was used for bioinformatic analysis (R Foundation for Statistical Computing, Vienna, Austria; version 3.5.3). Selection of miRs for normalization purposes was performed using geNorm [21] and NormFinder [22] algorithms. MiR with probable uniform expression levels in all study samples and a satisfactory abundance in CSF and PB was accepted as reference miR. Principal component analysis (PCA) with FactoMineR package [23] was used for exploratory multivariate data investigation in the discovery study. PCA helped distinguish between CSF samples with CNS^+^ or CNS^−^ background based on miRNomic profile and select candidate miRs for further examinations. Empirical Bayes statistical tests with false discovery rate (FDR) adjustment were performed using the limma package [24] for the evaluation of differential miR expression in the validation study. All linear models were adjusted for age at diagnosis and gender. To determine the diagnostic accuracy of promising markers, area under the receiver operating characteristic (ROC) curve and predictive values were defined by pROC package. Alpha level of 0.05 was used as criterion for statistical significance after FDR correction. The datasets used and analysed during the current study as well as full analysis code are available from the corresponding author on reasonable request.

## Results

### Differences in CSF microRNA expression between CNS^+^ and CNS^‒^ patients of the discovery cohort

Firstly, we examined whether microRNAs (miRs) were detectable in frozen cerebrospinal fluid (CSF) samples, collected from patients of the discovery cohort. Interestingly, in direct measurements with the use of a spectrophotometer, the concentration of unamplified cDNA prepared from total miR was slightly higher in CNS^‒^ patients than in the CNS^+^ group (mean ± standard error: 1222.3 ± 19.4 in CNS^−^ group vs. 1126.8 ± 9.1 in CNS^+^ group; p = 0.002).

**Fig. 1:**
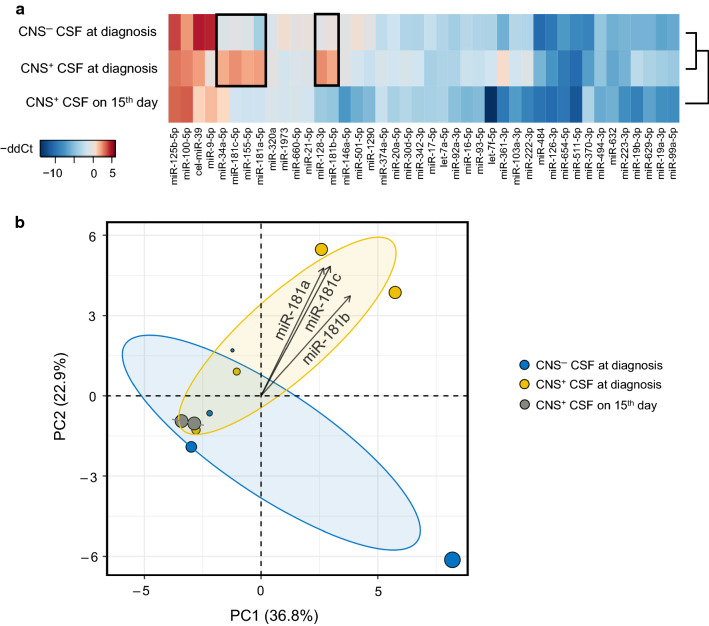
MiR-181-family has consistent contribution to altered microRNA expression in cerebrospinal fluid samples in discovery cohort. **a** Heatmap shows the overexpressed (red) and the downregulated (blue) microRNAs (miRs) in distinct sample types. Gross divergence in miR pattern of patients with or without initial CNS disease is highlighted with black frames. Mean relative expression levels were determined by ddCt algorithm, using miR-532-5p as reference miR and PB samples of control individuals as reference samples. **b** Biplot derived from principle component analysis shows clusters of CNS-positive and CNS-negative diagnostic samples at a confidence level of 95%. Corresponding loading plot (vectors) displays miR-181-family members. The cosine between two vectors approximates the correlation between miR variables. Position of vectors indicates considerable positive contributions to first and second principal component. *CNS* central nervous system, *CSF* cerebrospinal fluid, *ddCt* delta–delta Ct, *miR* microRNA, *PC* principal component

We used TLDA cards to screen the miR profile of CSF samples derived from patients with precursor B-cell ALL (pB-ALL) and peripheral blood (PB) samples of non-leukemic control individuals, respectively. In 83% of miRs, the standard error (SE) of the average Ct values in sample duplicates remained under 2 (range: 0.05‒4.69). Three (miR-106a-5p, miR-383-5p, miR-654-5p) of the 47 miRs were not detectable in any of the CSF samples. MiR expression measurements by TLDA cards identified miR-532-5p as the most stably expressed miR in collective examination of CSF and PB samples. Thus, miR-532-5p was assigned as reference miR and mean relative CSF gene expressions was calculated relative to the reference PB samples. All miRs were quantified at two different treatment checkpoints: (i) at diagnosis in both CNS^+^ and CNS^−^ patient groups and (ii) on the 15th follow-up day in CNS^+^ patients, see Fig. 1a.

Expression fold changes (relative to normalizer miR) of each miR in each sample were evaluated by principal component analysis (PCA) with a goal of identifying miRs that could potentially influence the classification of the patients by their CNS status. It was observed that three members of the miR-181-family (miR-181a-5p, miR-181b-5p, miR-181c-5p) had consistent and considerable positive contributions to all the first three principal components (PCs) and their aggregated contributions were above the expected average cut-off value (Additional file 1: Table S3). The first three PCs are included to reach a total sum of at least 70% of the original variation (PC1: 36.8%; PC2: 22.9%; PC3: 16.4%). The PCA biplot of PC1 and PC2 (Fig. 1b) shows the clusters of CNS^+^ and CNS^‒^ diagnostic samples with clustering performed at a confidence interval of 95%. Small angles between the miR-181-family members on loading plot of PC1 and PC2 (Fig. 1b) imply strong positive pairwise correlations.

A decrease in CSF miR levels to the 15th day of the treatment was also found in CNS^+^ patients. Among all tested RNAs, levels of miR-181a-5p, miR-181b-5p and miR-181c-5p showed the strongest reductions, as their expression fold changes (ΔFC) were 36.2, 299.4 and 39.8 times lower than at the diagnosis, respectively (see more information in Additional file 1: Table S4). In accordance with the previously mentioned results, miR-181a-5p and miR-181b-5p were selected for further validation.

### Diagnosis of CNS leukemia on the basis of miR-181a-5p level in CSF of patients with ALL

**Fig. 2:**
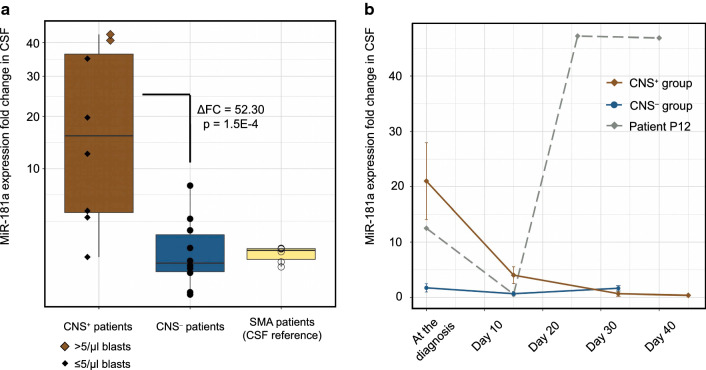
MiR-181a-5p level is a central nervous system involvement indicator in pediatric acute lymphoblastic leukemia. **a** Each dot indicates the miR-181a-5p expression of a patient. Horizontal line in box plots represents median fold change (FC), extent of boxes indicates upper and lower quartiles, whiskers show maximum and minimum values. **b** Lines are dedicated to follow the FC alterations in patient groups with and without meningeal leukemia and in a highlighted patient with progressive disease course during induction chemotherapy. Points indicate average FC values, while whiskers mark standard errors. *CSF* cerebrospinal fluid, *CNS* central nervous system, *SMA* spinal muscular atrophy, *ΔFC* difference in fold change

Relying on the TLDA card-based findings, two candidate markers (miR-181a-5p, miR-181b-5p) were tested using CSF samples in the validation cohort consisted of lymphoid, myeloid and mixed phenotype acute leukemia. We were able to confirm the high expression of miR-181a-5p in diagnostic CSF samples in a partly independent set of 8 CNS^+^ ALL patients compared with 10 CNS^−^ ALL patients by conventional qPCR, see Fig. 2a. However, similar results were not found when also including AML patients to the analysis. In those patients with ALL, miR-181a-5p expression levels conferred a more than 52-fold increased risk for CNS leukemia (CNS^+^ vs. CNS^−^ patients: ΔFC = 52.30, p = 1.49E−4). MiR-181a-5p relative expression level in CSF were independent of B- or T-cell immunophenotypes of ALL. Patients with overt CSF blastosis (CSF with a cell count of > 5/μl and blasts in excess on the cytospin slide) had the most outstanding miR-181a-5p expressions within the CNS^+^ ALL group. Median miR-181a-5p expression in ALL patients without CNS leukemia did not reach the median miR level detected in reference CSF samples collected from SMA patients (Fig. 2a). There were no significant expression differences in the case of miR-181b-5p in the validation cohort.

Regarding ALL patients in the validation cohort, the diagnostic efficacy of miR-181a-5p in CSF was evaluated by receiver operating characteristic (ROC) method. This novel marker yielded a sensitivity of 90.0% and specificity of 87.5% (area under the ROC curve, AUC: 92.5%) for CNS disease at a cut-off ΔFC value of 3.7, as shown in Additional file 2: Figure S1. To compare the clinical applicability of this miR marker with conventional cytomorphology technique, the latter one was also assessed based on previous reports [25] and signed as a ROC-curve in Additional file 2: Figure S1.

To address the issue of treatment-induced alteration dynamics of miR expression in time, we determined the miR-181a-5p expression level in CSF samples of ALL patients during the induction chemotherapy. MiR levels were measured at three timepoints: at diagnosis (before any drug administration), on the 15th day of therapy and on the 33rd day of therapy. We managed to obtain an extra sample from a patient (P2) with massive CNS involvement at 45th day of therapy and samples at exceptional therapy days (15th, 26th and 40th) from a patient (P12) with fatal disease course. Figure 2b shows changes in relative expression level of CSF miR-181a-5p in CNS^+^ and CNS^−^ ALL groups and, separately, in patient P12. The average level of miR-181a-5p decreased by 96.7% among CNS^+^ patients (P12 was excluded from this analysis) to the 33rd day of therapy, while it remained around the expression detected in diagnostic sample at our later time points in patients without CNS leukemia. Interestingly, patient P12′s miR-181a-5p expression has risen in parallel with clinically observed disease progression. Patient P12 could not finish the induction chemotherapy and was treated in intensive care unit until death. In a patient with CNS^+^ relapse (P1, ΔFC = 2.40) we observed a more than twofold rise in miR-181a-5p expression to the 15th treatment day from the initial level. This change was not seen in relapsed patients with blast-free CSF (P17 and P18; ΔFC = 0.06 and ΔFC = 0.83, respectively).

### Inferiority of bone marrow and peripheral blood miR-181-family expression to indicate CNS status in ALL

We studied whether the expression levels of miR-181a-5p and miR-181b-5p in PB and BM samples could provide diagnostic or predictive information on CNS leukemia involvement. Initial PB miR-181a-5p level significantly fell down to the 15th and to the 33rd day of treatment in ALL patients regardless of CNS status (to day 15: ΔFC = − 6.42, p = 0.02; to day 33: ΔFC = 7.95, p = 0.03). However, there was no difference in miR-181a-5p expression at diagnosis between CNS^+^ and CNS^‒^ ALL groups in PB.

MiR-181a-5p expression levels in BM highly exceeded corresponding CSF expression in CNS^‒^ ALL patients at the diagnosis (ΔFC = 21.97, p = 0.007), but there was no such relation in the CNS^+^ group. Initial BM expression of miR-181a-5p distinguished only those patients with CNS^+^ and CNS^−^ ALL who were characterized by precursor B-cell immunophenotype (ΔFC = 9.18, p = 0.04). However, miR-181a-5p level in PB of control individuals was lower than in BM of pB-ALL patients in both CNS status groups (CNS^+^ status: ΔFC = 55.82, p = 0.001; CNS^‒^ status: ΔFC = 6.08, p = 0.04). Decrease in miR-181a-5p level from the diagnosis of ALL to the 33rd day of therapy was also detectable in BM samples not only among CNS^+^ patients (ΔFC = − 187.96, p = 0.006), but also in the CNS^−^ group (ΔFC = − 8.54, p = 0.002).

### Atypical small extracellular vesicles in CSF of ALL patients with CNS involvement

**Fig. 3:**
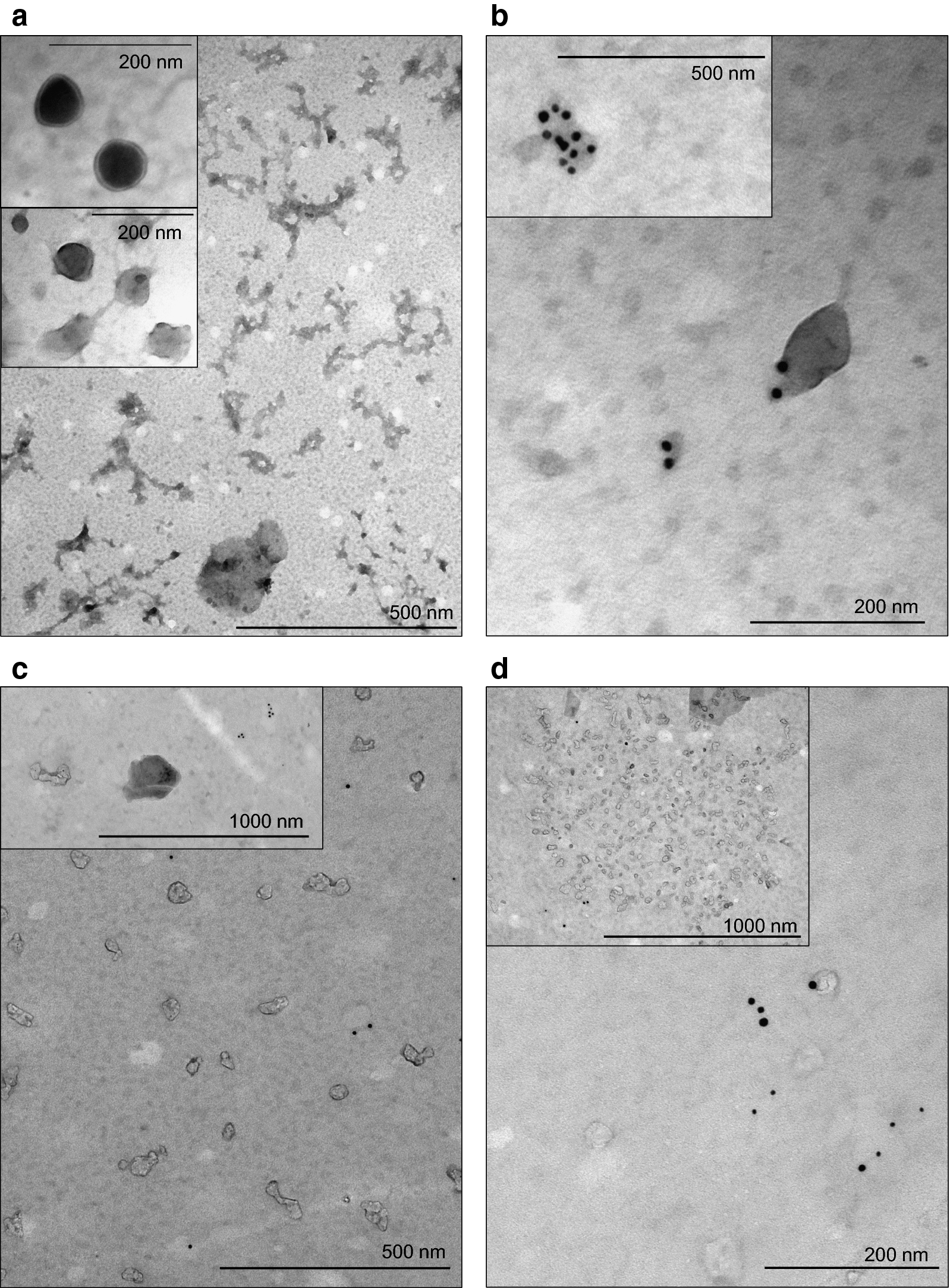
Atypical extracellular vesicles in cerebrospinal fluid of patients with different central nervous system (CNS) status. **a** Transmission electron microscopy (TEM) images of a cerebrospinal fluid (CSF) sample from a CNS^+^ patient with precursor B-cell acute lymphoblastic leukemia (pB-ALL) depict several small extracellular vesicles (EVs). Inserted panels show the absence of CD63/CD81 immunogold labelling which is a key feature of these EVs. **b** TEM images taken from the CSF sample of a patient with CNS^‒^ pB-ALL. Appearance of gold particles refer to CD63 (10 nm) and CD81 (5 nm) positivity of the EVs. **c** Small EVs in a patient with relapsed CNS^+^ T-cell ALL show mainly the absence of CD63/CD81 immunogold labelling. **d** CD63^+^/CD81^+^ small EVs in CSF from a patient with T-cell ALL without CNS involvement. Conversely, inserted slide shows a conglomerate of EVs without typical markers of exosomes originating from multivesicular body

Whether extracellular vesicle (EV) production from parameningeal leukemic infiltration can be demonstrated by CSF sampling is not yet known. Immunomagnetic bead separation technique was applied to compare the number of CD63^+^ particles (typically endosome-derived small EVs) in CSF samples of CNS^+^ and CNS^‒^ ALL patients (n = 2 and n = 2, respectively). Their measurable amount was very low and no difference was found in the proportion of CD63^+^ beads (mean ± SE: 1.0 ± 0.2% in CNS^+^ group vs. 1.1 ± 0.1% in CNS^−^ group). Next, from both CNS^+^ and CNS^−^ patient groups, a representative pB-ALL and a representative T-ALL CSF sample processed by ultracentrifugation (nonselective for cluster of differentiation markers) were supervised by immunolabelling for CD63 and CD81 markers using transmission electron microscopy (TEM). Interestingly, a considerable difference was observed in the density of vesicular elements between CNS^+^ and CNS^−^ patients, as shown in Fig. 3. High amount of EVs in the CNS^+^ pB-ALL sample mostly showed CD63^‒^/CD81^−^ characteristics (Fig. 3a). Whilst, there were low number of EVs in the CNS^−^ pB-ALL sample (Fig. 3b). CSF samples of T-ALL cases showed quite similar pattern, but in this immunophenotype group we found a CD63^−^/CD81^−^ conglomerate of EVs in the CNS^−^ sample as well (Fig. 3c, d). Presence of CD63^+^ and CD81^+^ small EVs in CSF, even if a low number, were confirmed in both CNS groups and immunophenotypes.

## Discussion

Contrary to the overall survival rate of 70–85% among children with acute leukemia, leukemic infiltration of the central nervous system (CNS) niche is associated with relapses and major therapeutic obstacles [2]. We still lack methods to follow up CNS leukemia with appropriate sensitivity. There are two major clinical enigmas related to CNS disease: (i) how can we better screen occult meningeal leukemia and thereby prescribe adequate CNS-directed treatment for those who need it, and (ii) may we apply novel molecularly targeted therapies against CNS infiltration in the era of precision oncology? It is also not fully understood whether the initial CNS leukemia burden or its dynamics during treatment have more prognostic value.

Here we proposed a potential biomarker for the CNS status in ALL. Cerebrospinal fluid (CSF) miR-181a-5p expression levels were unexpectedly high in patients with cytologically confirmed CNS^+^ ALL, while levels in those lacking meningeal involvement were measured around control levels. This result originated from a discovery screening of 47 candidate miRs and was validated in an extended, but still limited study set. Similarly, a considerable difference was observed in the density of small vesicular elements in CSF samples of CNS^+^ and CNS^‒^ patients with ALL. To date, only one study has reported that miRs can be measured in the CSF collected from children with leukemia [26]. Furthermore, our study is unique in that miR expression alterations can be followed individually in time thanks to the serial sample collection, and miR patterns in different compartments of sampling can be compared. CSF-based EV studies in leukemia are also in their early infancy.

Despite the omnipresence of ribonucleases, miRs were found to be highly stable in all body fluids, hence, could be utilized as clinical biomarkers [27]. MiR-181a serves as a regulator of normal hematopoiesis and its disruption has been linked to various types of cancer, including hematological malignancies [28]. It is one of the most abundant miRs in lymphocytes [29]. In acute leukemia, the exact role of miR-181a is still unclear as contradicting papers claim its oncogene as well as tumor suppressor effects [30–33]. Besides, several mechanisms spreading to the CNS by ALL cells have been revealed recently and can be influenced by miR-181a regulatory actions. Of particular interest to our findings, overexpression of vascular endothelial growth factor A (*VEGFA*), a miR-181a regulated hypoxia-responsive gene, proved to provide survival advantage to CNS-penetrating ALL cells in the hypoxic, nutrient-poor microenvironment of the CNS. This phenomenon was described in both primary pre-B ALL cells isolated from the CSF of children with CNS involvement and a mouse primograft model for CNS^+^ ALL [34–36]. The positive correlation between the miR-181a expression and VEGFA production in a non-epithelial tumor tissue was proposed through the scrutiny of human chondrosarcoma cells [37]. A detailed explanation how miR-181a promotes the SRC/VEGF signaling pathway was shown in preclinical models of colorectal cancer [38]. A similar mechanism might work in ALL, too. Yet, the nature of CNS disease evolution is not fully understood. While the VEGFA mechanism suggests increased vascular permeability and reduced barrier function of endothelium to make cell migration possible into the CNS, a novel study claims that the whole process happens abluminally, along emissary bridging vessels based on integrin alpha-6 (ITGA6)-laminin interaction [39]. Interestingly, *ITGA6* may interact with miR-181a according to the mirDIP v4.1 integrative miR target predictor database [40].

Whether the ability to invade the CNS is a generic property of all ALL blasts or only a subpopulation of cells can selectively enter this compartment is a subject of recent debate. We found that bone marrow (BM) miR-181a-5p expression of CNS^‒^ patients (i) exceeded the average level of CNS^−^ CSF and control samples and (ii) responded to the chemotherapy with significant reduction, similarly to the CNS^+^ BM samples. Thus, our data tend to support the theory that ALL cells in the BM compartment have a common ability to reach meningeal surfaces, as previously proposed [41, 42]. Also, the seeding of CNS leukemia may depend on a dynamic relationship between extramedullary microenvironment and blast cells which is influenced by regulatory elements (e.g. miR-181a), rather than predetermined cell-intrinsic and clone-specific ‘CNS-tropic’ features (e.g. cell surface protein composition). Basically, it was out of our aims to identify the cellular origin of miR-181a-5p in this study setting. Yet, there are some points which suggest the examination of leukemic blasts as potential miR-181 secretors: (i) miR-181a is a well-known small RNA produced by lymphoid cells [29]; (ii) miR-181a was found in significant amount in blast-rich BM samples, regardless of CNS status; (iii) in CNS^−^ CSF samples, where blasts were absent, the average expression of miR-181a was very low. However, it is also a possibility that miR-181a originates from non-leukemic cells communicating with leukemic blasts (e.g. stromal cells in BM or resident cells in CNS leukemic niche) and we measured this phenomenon in our samples. There are plenty of evidence that miR-181-family plays role in the modulation of tissue differentiation, remodeling and degeneration in the CNS, moreover, it has anti-inflammatory effect in the context of neuroinflammation [43].

According to our data, miR-181a-5p may provide about 35% sensitivity benefit compared to the cytospin method when diagnosing ALL infiltration in the CNS at the initiation of chemotherapy. However, a paper described that occult meningeal disease during therapy resulted in significantly higher rates of relapses and death in pediatric ALL, while no such influences were found with initial CSF evaluation [44]. Thus, the alteration of initial miR-181a-5p level might be considered to estimate prognosis.

Candidate miR expression analysis of peripheral blood (PB) samples identified no benefit of measuring miR-181-family levels to diagnose CNS involvement. In contrast, we showed the possible role of diagnostic BM miR-181a-5p expression in determining CNS status, however, it was demonstrated solely in pB-ALL patients. This finding might propose a less invasive alternative to LP as BM aspiration which is sometimes also performed at CSF sampling time points. However, higher BM miR-181a-5p level may be associated with general miRNomic disturbance under leukemic conditions as CNS^‒^ patients showed elevated relative expression as well. We rather prefer the investigation of miR-181a-5p level alteration in time as it may monitor the CNS-attacking blast capacity of the BM compartment. To indicate subsequent CNS relapse by BM examination, others identified cellular proteins [45] and miR pattern [46] in BM cells of ALL patients, but venipuncture-based CNS involvement markers were not published.

This is the first study in which small extracellular vesicle (EV) composition of CSF was assessed in relation with CNS disease state in acute leukemia patients. Atypical small EVs were found in unexpectedly high density in CNS^+^ diagnostic CSF samples. The phenotype of these vesicles is not fully understood. They were negative for CD63 and CD81, which contradict the multivesicular body origin [47]. Nonexosomal subpopulation of small EVs was previously demonstrated [48], and most of the EVs in CNS^+^ CSF reached the lower limit of the microvesicle size range. Otherwise, it is unlikely that these bodies are lipoproteins with EV-like appearance as CSF lipoprotein content is minimal [49].

In summary, our data represent the first description of a miR as possible CNS infiltration marker in pediatric ALL and extends our knowledge about a small EV subgroup that may associate with CNS^+^ status. However, several limitations should be noted when interpreting our results. Since CNS involvement is a rare condition in childhood leukemia, the difficulty of obtaining CNS^+^ samples resulted in low patient numbers. Our results should be verified in larger cohorts before concluding any practical benefit. Another bias is the selection of patients with and without the meningeal disease, which relied on the insensitive conventional cytologic assessment tools. Yet, this study provides the rationale for future investigations of miRs in the CSF for diagnostic and prognostic purposes. Our results encourage further research regarding the targeted therapy of CNS leukemia [35, 36] or miR-181a-based therapeutics as seen in lung cancer models in vitro [50].

## Conclusions

Disease course indicators are the “Achilles heel” of therapy success in pediatric ALL and miR-181a-5p expression in CSF may provide potential new tools for this purpose. Based on our experiments, it can worth to further examine miR-181a-5p level in CSF as highly sensitive and specific CNS involvement indicator in ALL to find a novel approach which can upgrade contemporary diagnostic methods. Moreover, with our very first experiments, we propose further CSF small EV measurements and characterization from CNS leukemia perspective.

## Supplementary information

**Additional file 1: Table S1.** Detailed description of patient cohorts. **Table S2.** List of 47 microRNAs screened in discovery study. **Table S3.** Contribution of miR-181-family members to principal components. **Table S4.** Rate of expression decrease of microRNAs in discovery cerebrospinal fluid samples at day 15^th^.

**Additional file 2: Figure S1.** Comparison of receiver operating characteristic (ROC) curves of miR-181a-5p and conventional cytospin methods.

## Data Availability

The datasets used and/or analysed during the current study are available from the corresponding author on reasonable request.

## Abbreviations

ALL: Acute lymphoblastic leukaemia
AML: Acute myeloid leukaemia
BM: Bone marrow
CNS: Central nervous system
CSF: Cerebrospinal fluid
Ct: Cycle threshold
EV: Extracellular vesicle
FDR: False discovery rate
LP: Lumbar puncture
miR: microRNA
MPAL: Mixed phenotype acute leukaemia
PB: Peripheral blood
PCA: Principal component analysis
PFP: Platelet-free plasma
qPCR: Quantitative polymerase chain reaction
ROC: Receiver operating characteristic
SMA: Spinal muscular atrophy
TEM: Transmission electron microscopy
TLDA: Custom TaqMan Advanced Low-Density Array
VEGFA: Vascular endothelial growth factor A
ΔFC: Expression fold change
