# Supplementary material for: MicroRNA-181a as novel liquid biopsy marker of central nervous system involvement in pediatric acute lymphoblastic leukemia
Source: J Transl Med. 2020 Jun 22;18:250. doi: 10.1186/s12967-020-02415-8 (PMC7310470; doi:10.1186/s12967-020-02415-8)
Supplement: Supplementary file 2 — Additional file 2: Figure S1. Comparison of receiver operating characteristic (ROC) curves of miR-181a-5p and conventional cytospin methods. [file 12967_2020_2415_MOESM2_ESM.pdf]

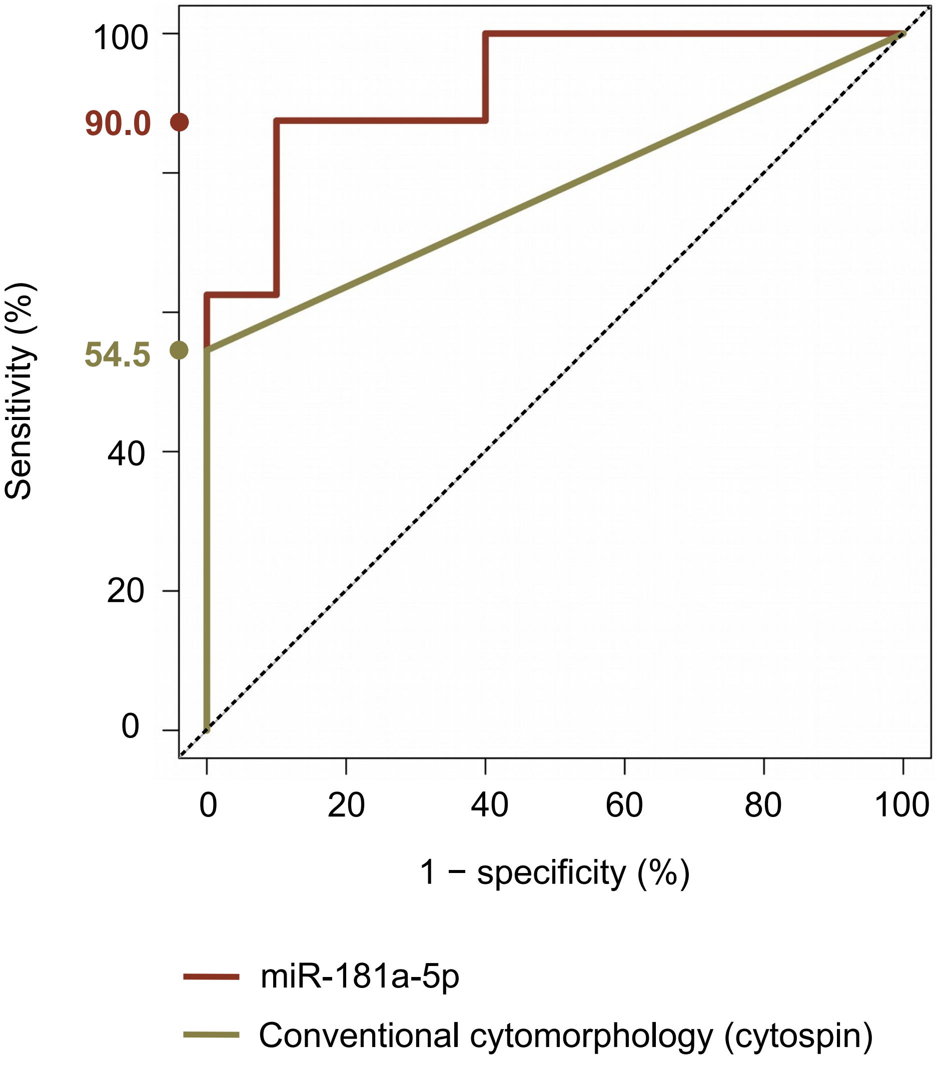


**Figure S1.** Comparison of receiver operating characteristic (ROC) curves of miR-181a-5p and conventional cytospin methods. Curve of conventional cytomorphology was produced based on information found in the literature (reviewed in de Graaf et al. 2011), where diagnostic efficacy of cytomorphology was assessed being aware of flow cytometry data.
